# Editorial for the “Green Chemistry” Section in the Journal Molecules: Focus on Solvents

**DOI:** 10.3390/molecules25215151

**Published:** 2020-11-05

**Authors:** James Sherwood

**Affiliations:** Green Chemistry Centre of Excellence, University of York, Heslington, York YO10 5DD, UK; james.sherwood@york.ac.uk

It is a pleasure to write this editorial highlighting some of the recent papers discussing solvents in the Green Chemistry section of *Molecules*. Fundamentally, the function of a solvent is to dissolve substances, as is important for extractions and formulation science. Solvents also control the rate and selectivity of reactions as well as chemical equilibria. It is for these reasons that solvents are widely used, and they have a pivotal role in the transition to a sustainable chemical industry. However, solvents represent the majority of the waste created by many processes and formulated products, may contribute to air pollution, and frequently possess a number of human health and safety hazards.

Alternative solvents must be developed and used in such a way to alleviate the problems of conventional solvents. This requires an understanding of the properties of new solvents, including their health, safety, and environmental hazards. Tightening chemical regulation and the desire for a lessened dependency on fossil feedstocks encourages the development of bio-based solvents. Samoilov and co-workers report the synthesis and comprehensive characterisation of cyclic ketals and glycol ethers from bio-based diols [1]. The question of identifying appropriate applications for new solvents has been addressed by Sels et al. [2]. Artificial Intelligence (AI) is capable of collating molecular property data into clusters of similar solvents. This can be used to identify potential substitutions in favour of safer solvents. This methodology has been tested on a diverse set of applications, from organic synthesis to formulations.

The past two years have seen a considerable increase in the number of papers accepted into the Green Chemistry section of *Molecules*. The majority of papers document the use of solvents to some degree, as the medium for reaction chemistry, in order to conduct chromatographic separations, to extract and prepare analytical samples, or to fabricate materials. Contributions primarily dedicated to solvent properties and their applications accounted for about one in every seven published works in the Green Chemistry section of *Molecules* between 2019 and 2020 (to date). The two most popular subjects have been the extraction and fractionation of biomass, and the analysis of food and environmental samples. I think it is helpful for potential authors to recognise that in many of the journals that cover general green chemistry, contributions to organic synthesis and materials science usually outweigh the analytical sciences. This means that *Molecules* has a somewhat privileged role—highlighting solvent applications relating to extraction and analysis [3,4,5,6,7,8,9], yet performed in a way which is environmentally acceptable.

The representation of different types of solvents in *Molecules* is swayed by the Special Issues that currently open. Nevertheless, over the last few years, the number of studies about ionic liquids, deep eutectic mixtures, and supercritical fluids appearing within the Green Chemistry section have been approximately equal. These categories of solvents all provide a degree of tunability, be it the exchange of ions to produce countless ionic liquids, changing the ratio of components in deep eutectic mixtures, or adjusting the density of a supercritical fluid through pressure and temperature to optimise the selectivity of extractions. The ability to fine-tune and modify these solvents offers immense possibilities that continue to be explored.

The use of supercritical carbon dioxide (scCO_2_) as a solvent is intriguing because of the enhanced diffusivity of supercritical fluids compared to liquids. This can be exploited to perform extractions from solid matrices such as biomass. It is also possible to impregnate bio-composite polymers with active ingredients. Pajnik et al. have recently produced starch–chitosan films containing the natural antimicrobial agent thymol using this technique [10].

Deep eutectic mixtures permit the use of renewable compounds to create highly polar solvents (in many ways the opposite of scCO_2_). These solvents have found use in a variety of applications, one being the fractionation of biomass. Canela-Garayoa and co-workers have shown the separation of lignocellulosic food processing waste can be performed with a deep eutectic solvent made by transforming glycerol into a quaternary ammonium salt and combining with lactic acid [11]. The recovery of lignin was superior compared to the analogous choline chloride–actic acid deep eutectic solvent.

Hydrophobic deep eutectic mixtures have been used to extract compounds from samples for the analysis of contamination and impurities. A deep eutectic mixture of decanoic acid and tetrabutylammonium bromide was used by Kachangoon et al. to determine the presence of neonicotinoid insecticides in soil, water, and food samples [12]. The extracted pesticides were analysed by HPLC with detection limits accurate enough to confirm conformity with regulated limits.

Contributions to *Molecules* are welcome from wide range of disciplines within the chemical sciences. The Green Chemistry section of *Molecules* encourages submissions discussing alternative solvents (bio-based solvents, supercritical fluids, ionic liquids, deep eutectic mixtures etc.), organic chemistry in green solvents, extractions and processing, and green analytical chemistry, but this is not a definitive list and any green chemistry research is considered.
